# Leukocyte Populations and C-Reactive Protein as Predictors of Bacterial Infections in Febrile Outpatient Children

**DOI:** 10.4274/Tjh.2013.0057

**Published:** 2014-03-05

**Authors:** Zühre Kaya, Aynur Küçükcongar, Doğuş Vurallı, Hamdi Cihan Emeksiz, Türkiz Gürsel

**Affiliations:** 1 Gazi University Medical School, The Pediatric Hematology Unit, Department of Pediatrics, Ankara, Turkey

**Keywords:** Leukocyte populations, C-reactive protein, Febrile children

## Abstract

**Objective:** Infections remain the major cause of unnecessary antibiotic use in pediatric outpatient settings. Complete blood count (CBC) is the essential test in the diagnosis of infections. C-reactive protein (CRP) is also useful for assessment of young children with serious bacterial infections. The purpose of the study was to evaluate leukocyte populations and CRP level to predict bacterial infections in febrile outpatient children.

**Materials and Methods:** The values of CBC by Cell-DYN 4000 autoanalyzer and serum CRP levels were evaluated in 120 febrile patients with documented infections (n:74 bacterial, n:46 viral) and 22 healthy controls.

Results: The mean CRP, neutrophil and immature granulocyte (IG) values were significantly higher in bacterial infections than in viral infections and controls (p<0.05). C-reactive protein was significantly correlated with neutrophil level in bacterial infections (r: 0.76, p<0.05). Specificity of IG was greatest at 93%, only a modest 56% for neutrophil and mild 18% for CRP, whereas 100% for combination of IG, neutrophil and CRP.

**Conclusion:** Acute bacterial infection seems to be very unlikely in children with normal leukocyte populations and CRP values, even if clinically signs and symptoms indicate acute bacterial infections.

## Abstract (tr)

**Amaç:** Enfeksiyonlar pediatri polikliniklerinde gereksiz antibiyotik kullanımının en önemli nedeni olmaya devam etmektedir. Tam kan sayımı (CBC) enfeksiyonların tanısında kullanılan önemli bir testtir. C-reaktif protein (CRP) ise ciddi bakteriyel enfeksiyonu olan küçük çocukların değerlendirilmesinde yararlıdır. Bu çalışmanın amacı polikliniğe başvuran ateşli çocuklarda bakteriyel enfeksiyonu ayırdetmede CRP düzeyi ve lökosit popülasyonunun önemini değerlendirmektir.

**Gereç ve Yöntemler:** Polikliniğe başvuran 120 ateşli çocukta Cell-DYN 4000 ile analiz edilen CBC değerleri ile CRP düzeyi, 74 bakteriyel, 46 viral ve 22 kontrol grubunda değerlendirildi.

**Bulgular:** Ortalama CRP, nötrofil ve immature granulosit (IG) değerleri bakteriyel enfeksiyonlarda, viral enfeksiyon ve kontrol grubuna göre anlamlı yüksekti (p<0,05). Bakteriyel enfeksiyonlarda CRP ve nötrofil değerleri arasında anlamlı ilişki bulundu (r:0,76, p<0,05). Özgüllük IG için %93 ile en yüksek, nötrofil için %56 ile orta ve CRP için %18 düşük düzeyde bulunmasına rağmen IG, nötrofil ve CRP kombinasyonu için %100 bulundu.

**Sonuç:** Çocuklarda klinik belirti ve bulgular akut bakteriyel enfeksiyonu işaret etse bile, normal lökosit popülasyonu ve CRP değeri olan hastalarda akut bakteriyel enfeksiyon olasılığı düşüktür.

## INTRODUCTION

Despite rapid improvement in health care over the past decades, fever continues to be a major cause of admissions, laboratory work-up and antibiotic uses in pediatric outpatient settings (POS) [1,2,3,4]. Fever due to viral infections can be particularly difficult to distinguish from that in children with clinical signs of bacterial infections [5]. 

To date, C-reactive protein (CRP) has been used to make distinction between bacterial and viral infections but it has been reported as neither sensitive nor specific enough for bacterial infections [6,7]. One available strategy is to monitor changes in leukocyte populations and CRP associated with the host response to pathogens [8,9]. However, these markers have mostly been studied in infants and younger children below 3 years of age and for serious bacterial infections excluding older children and localized bacterial infections [10,11,12]. Furthermore, novel hemogram analysers allow reliable measurement of a broad panel of complete blood count (CBC) parameters. The variant lymphocyte (VL) and immature granulocyte (IG) parameters have become increasingly popular. Usefulness of monitoring VL and IG for identifying infectious process have been examined in a few studies [13,14,15,16]. However, its usefulness remains controversial in children. The aim of this study was to evaluate the usefulness of CRP and leukocyte populations as early diagnostic markers of bacterial infections in febrile outpatient children.

## MATERIALS AND METHODS

Data were collected from 120 consecutive children who presented with fever and 22 children with age matched healthy controls during two months period at Pediatric Outpatient Clinic of Gazi University Hospital. Patients were eligible to participate if they had a clinical and/or radiological and/or microbiological diagnosis of viral or bacterial infections confirmed by residents in Department of Pediatrics and decision for antibiotic treatment was made in accordance with the management of infection guidance for primary care at www.hpa.org.uk web page [17].

The inclusion criteria were: 1) age between 2 and 18 years, 2) fever was defined as an axillary temperature above 38o C, as used for the diagnostic criteria of febrile episodes in the past 24 hours. We excluded infants <2 years, children requiring hospitalization for fever, ongoing antibiotic treatment at the time of evaluation and specific chronic conditions.

Informed consent and approval by our Institutional Review Board were obtained. 

**Laboratory Analysis**

Venous blood samples for complete blood count (CBC) were collected into vacutainer tubes containing K2EDTA (Becton Dickinson, New Jersey, USA) and analysed by Cell-DYN 4000 Hematology Analyzer (Abbott Diagnostics, Santa Clara, CA) within 6 hours of admission. The upper limit of the reference interval for leukocyte populations was described according to the age (Table 1) (18). Serum level of CRP was measured using a nephelometric assay (Specific Protein Analyser, Beckman, Marburg, Germany) with the normal range as 0 to 6 mg/L. The abnormal values for CRP and leukocyte populations were regarded as above the upper normal limit of the reference interval. The microbiological tests including viral serology for EBV, CMV, HSV, Parvo-B19, urine, throat and stool cultures were retriewed from Hospital Information System. All radiological tests were evaluated by expert radiologists.

**Statistical Analysis**

Statistical analysis was performed using SPSS 15.0 (SPSS Inc., Chicago, IL). Data was expressed in mean±SD. All the categorical variables were calculated using Chi-square analysis. Different groups of patients were compared using the Mann-Whitney U test and correlations were calculated by Spearman’s correlation test. Wilcoxon test was used to evaluate within the group comparison. Parameters considered significantly associated with a high risk of bacterial infections were selected in univariate analysis, and the logistic regression for multivariate analysis was calculated for odds ratio (OR) and 95% confidence interval (CI). P values <0.05 were considered to be statistically significant.

## RESULTS

Demographic and clinical characteristics of patients are summarized in Table 1. Of the 120 children, 76 (63.4%) were boys and 44 (36.6%) were girls. The median age was 5 years (2-18 years). There was no difference between bacterial and viral pathogens in respect to mean age and gender (p>0.05). 

**Infection Types and Locations**

The most common primary sites of infection in order of frequency were ear-nose-throat, lung and urinary tract system. Seven (5.8%) of the 120 children had definitive and 67 had (56.0%) probable bacterial infections. Probable viral infections comprised 44 patients (36.6%) with upper respiratory tract infections along with flu-like symptoms and negative bacterial markers (no viral cultures were performed), one patient with documented EBV, and one with Parvo-B19 (1.6%). 

**CBC and CRP Levels **

The mean levels of CRP and the leukocyte populations are demonstrated in Table 2. At the time of diagnosis, patients with bacterial infections had increased serum CRP level, neutrophil, lymphocyte, and monocyte percent compared with the control group (p<0.05). 

The mean level of serum CRP, neutrophil and IG levels showed significant decrease two weeks after the start of antibiotic treatment as compared to their baseline at diagnosis (p<0.05). Elevated CRP and neutrophil levels were noted in only 27 of 67 patients with probable bacterial infections who were treated with antibiotics. 

Univariate analysis showed that a high levels of CRP and neutrophils were associated with bacterial infections, while VL positivitiy was more diagnostic for viral infections in febrile children. In multivariate analysis, CRP was a better indicator for bacterial infections with an odds ratio of 6.1 (95% CI, 1.5-24.6 ) (Table 3).

The specificity of neutrophil and IG levels was higher for diagnosing bacterial infections than that of CRP alone. However, the combination of CRP, neutrophil and IG levels had the highest specificity for predicting bacterial infections. Normal values for neutrophil, IG and CRP excluded bacterial infections had a 100% specificity and positive predictive value in a generic context. Variant lymphocyte and lymphocyte levels were found highly specific and statistically superior to CRP in viral infections (Table 4).

**Figure 1:**
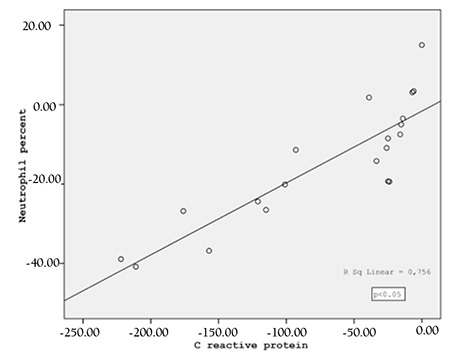
Correlation between changes in C reactive protein and neutrophil percent

There was a significant positive correlation (r:0.76, p<0.05) between CRP and neutrophil level in the bacterial infections while there was a significant negative correlation between CRP and lymphocyte (r:-0.75, p<0.05) for viral infections (Figure 1). 

## DISCUSSION

Despite viral infections represent the most frequent outpatient infections in children, the increasing rates of antibiotic resistance due to unnecessary antibiotic use have become a major threat for child health [1,2,3,4]. Therefore an immediate and appropriate strategy for this population in POS is required to prevent unnecessary antibiotic use and to reduce treatment delays. In this study, we analysed 120 febrile patients in POS and validated the diagnostic value of CRP and CBC for infectious complications in children.

The early diagnosis of bacterial infections in patients with fever is challenging [19,20,21]. Focus of infection is uncertain, and only a few clinical signs such as tonsillopharyngitis, otitis and sinusitis may indicate bacterial infections in many cases of fever. Although cultures are the gold standard for the diagnosis of bacterial infections, sampling and testing is time consuming and their results are not immediately available. Therefore, a predictive tool to diagnose bacterial infections in fever is crucial for early diagnosis and treatment in POS.

Several inflammatory markers have been studied for the diagnosis of infections. Among them, CRP is frequently used and is a good marker for infection [22,23,24,25]. Few data are available evaluating CRP for the detection of bacterial infections in children with fever [25,26,27]. The prevalance rate of bacterial infections varies between 28% and 82% in febrile children seen at the emergency departments. In the present study, we have found the prevalance of proven and probable bacterial infections to be 62% in children with fever who started antibiotic treatment in POS. So far, the association between CRP and bacterial infections was only evaluated in the hospitalized children or at the emergency department and predominantly in younger children below three years of age [6,7,8,9,10,11,12,25,26,27,28]. 

Most authors concluded that CRP >40 mg/dL indicates severe bacterial infections [6,9,22]. We also found that the bacterial infections had higher mean CRP levels compared with the viral infections in POS. However, a multivariate model showed that CRP was the only independent variable for the association between viral and bacterial infections. More recently, several authors have reported the quantitative evaluation of CRP as a diagnostic marker of bacterial infections, sensitivity and specificity ranging from 57% to 100% and from 50% to 100%, respectively [6,7,8,9,25]. In our study, CRP was found to be a highly (89%) sensitivite but has quite low (18%) specificity.

It has been widely accepted that CRP was not elevated during viral infections. We found that CRP was weakly positive in only 3 of 16 patients with viral infections. It is possible that there may be coexisting latent bacterial infections in these patients. Thus, CRP positivity alone may not be helpful to estimate the causative pathogens particularly in febrile patients. Other authors have described “unconventional” inflammatory markers such as procalcitonin, interleukin 6, which have been used as research tools but not achieved widespread acceptance in routine practice [22,23,24]. 

Some laboratory routine tests such as CBC are fast, economical, and universally available, and often aid primary clinicians with decision making about patients with suspected bacterial infections [29,30]. Thus the rapid availability of the results of CBC as well as CRP could provide considerable advantage for both patients and clinicans. In the present study, we have used Cell-DYN 4000 device which enabled us simultaneously measure several different CBC parameters. As in other studies, our results showed a significant positive correlation between CRP and neutrophil levels in supporting of bacterial infection. On the other hand, in patients with viral infections, lymphocyte showed negative correlation with CRP level (r:-0.75, p<0.05) [24]. The suggested cut-off values of <5% variant lymphocyte excluded viral infections with the 96% specificity (Table 4).

Our results have shown that normal neutrophil and IG levels and CRP value excluded bacterial infections with a predictive value of 100% in children presenting with fever. Antibiotics should not be recommended in such cases with a normal neutrophil and IG levels and CRP value, even when bacterial infections is suspected clinically. If typical signs and symptoms of acute bacterial infections continue and abnormal leukocyte populations and/or CRP value increases above the upper limit of the reference interval, the patient should be treated by antibiotic. Otherwise, continued observation is recommended. A single CRP test will not be very indicative of bacterial infection but a combination of neutrophil, IG and CRP may provide more valid information considering the complex relationship between the antibiotic use and the clinical features of bacterial infection. To our knowledge, there are no previous studies relating measurement of IG and VL in febrile outpatient children. Although our results should be confirmed in a prospective study including larger number of patients, we believe that IG, neutrophil and CRP values may guide physicians to make a distinction between viral and bacterial infections.

In conclusion, neutrophil and IG levels together with CRP constitute a rapid and cheap diagnostic tool with moderate diagnostic value in children with bacterial infections. Using these laboratory test, physicians can avoid unnecessary antibiotic use in approximately two thirds of children with suspected bacterial infections in POS.

## CONFLICT OF INTEREST STATEMENT

The authors of this paper have no conflicts of interest, including specific financial interests, relationships, and/ or affiliations relevant to the subject matter or materials included

## Figures and Tables

**Table 1:**
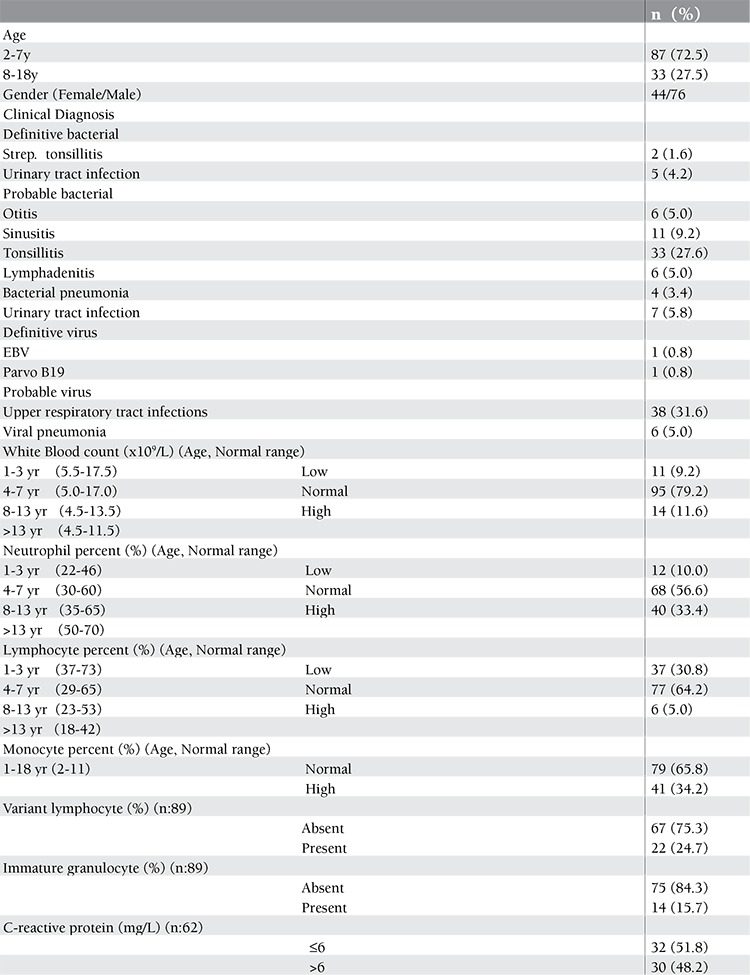
Demographic features and clinical characteristics of patients.

**Table 2:**
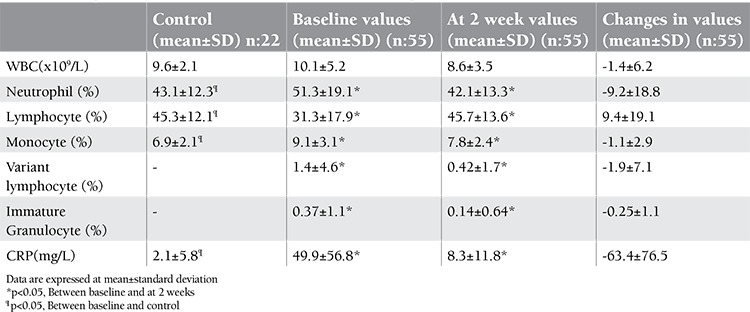
Changes in variables from baseline to two weeks following antibiotic treatment.

**Table 3:**
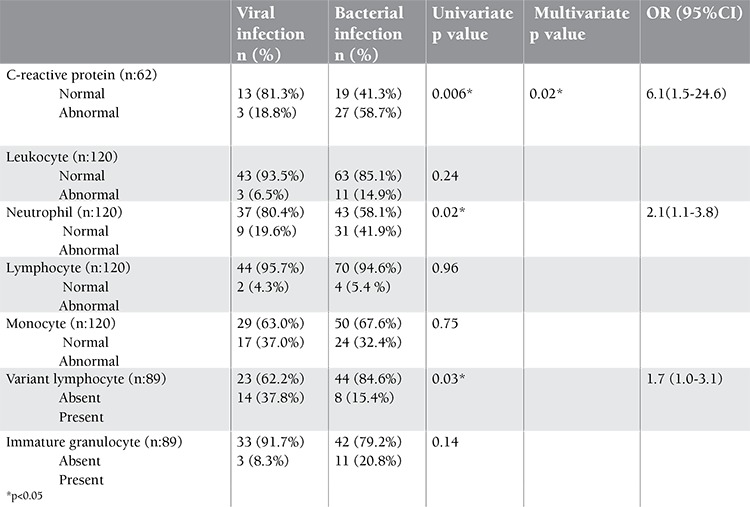
C-reactive protein and leukocyte populations in relation to the infections using by univarite and multivariate analysis.

**Table 4:**
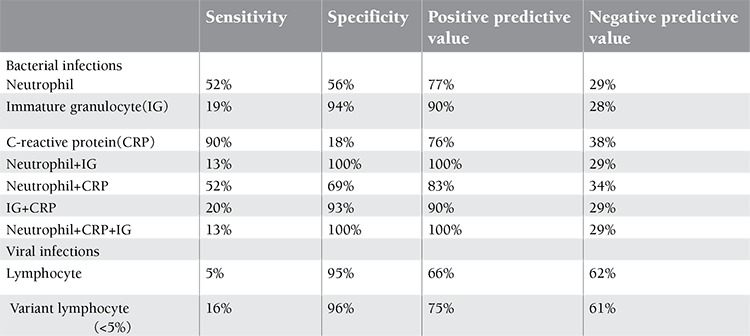
Diagnostic accuracy of C-reactive protein (CRP) and leukocyte populations in viral and bacterial infections.
